# Genomic Characterization of NDM-1 Harboring Extensively-Drug Resistance *Klebsiella pneumoniae* Isolate From ICU-Admitted Patient With COVID-19

**DOI:** 10.1155/jotm/6616950

**Published:** 2025-04-14

**Authors:** Abolfazl Rafati Zomorodi, Himen Salimizand, Niloufar Mohseni, Maryam Hafiz, Helia Nikoueian, Tahereh Gholamhosseini-Moghaddam, Fatemeh Aflakian

**Affiliations:** ^1^Department of Bacteriology and Virology, School of Medicine, Shiraz University of Medical Sciences, Shiraz, Iran; ^2^Vaccine and Infectious Disease Organization, Saskatoon, Saskatchewan, Canada; ^3^Department of Vaccinology and Immunotherapeutics, School of Public Health, University of Saskatchewan, Saskatoon, Saskatchewan, Canada; ^4^Department of Pathobiology, School of Veterinary Medicine, Ferdowsi University of Mashhad, Mashhad, Iran; ^5^Department of Chemical Engineering, Faculty of Advanced Technology, Quchan University of Technology, Quchan, Iran

**Keywords:** bla_NDM−1_, carbapenemase, *K. pneumoniae*, SARS-CoV-2, XDR

## Abstract

Currently, carbapenem-resistant *Klebsiella pneumoniae* (CR-KP) strains, particularly those producing New Delhi metallo-beta-lactamase (NDM), are increasingly recognized as a significant threat to global health. The present study aimed to conduct a genomic analysis of an NDM-1-producing CR-KP strain isolated from patients with coronavirus disease of 2019 (COVID-19) admitted to the intensive care unit (ICU). The *K. pneumoniae* isolate was obtained from the bronchoalveolar lavage fluid of a 68 year-old male patient hospitalized in the ICU with COVID-19 at Besat Hospital in Sanandaj, Iran. The minimum inhibitory concentrations (MICs) for 15 antibiotics were determined using the VITEK 2 system. Genomic analysis of the isolate was performed using whole genome sequencing. The CRKP-51 strain was identified as an extensively drug-resistant (XDR) strain, exhibiting resistance to all tested antibiotics except tigecycline (MIC = 2 μg/mL). The highest resistance values were recorded against sulfamethoxazole-trimethoprim (SXT), nitrofurantoin (NIT), and piperacillin-tazobactam (TZP), with MICs of ≥ 320, 256 μg/mL, and ≥ 128 μg/mL, respectively. Multilocus sequence typing revealed that CRKP-51 belonged to sequence type 15 (ST15). The IncHI1B replicon type associated with this strain harbored several resistance genes, including *bla*_*NDM*−1_*, armA*, *msrE*, *mphE*, *BRP* (MBL), *bla*_*OXA*−1_, *aadA*2, *dfrA*12, *qnrB*1, *bla*_*CTX*−*M*−15_, and *cat*1. High-risk *K. pneumoniae* clones, such as ST15, are increasingly associated with antimicrobial resistance and the emergence of XDR strains in ICUs. Additionally, the global dissemination of the NDM enzyme occurs through various plasmid replicon types. Therefore, monitoring local epidemiology is essential for the effectiveness of antimicrobial stewardship programs.

## 1. Introduction

Nowadays, *Klebsiella pneumoniae* (*K. pneumoniae*) is mainly taken attention as one of the significant bacteria involved in antibiotic resistance issues; especially carbapenem-resistance *K. pneumoniae* (CR-KP) strains [1]. Carbapenems are often used as a last resort for treating infections caused by multidrug-resistant (MDR) *K. pneumoniae* isolates. Consequently, the sharp increase in CR-KP isolates has been identified as a significant global health concern by the World Health Organization (WHO) [2, 3].

Hydrolyzing most β-lactams (except aztreonam, a monobactam), not inhibited by available β-lactamase inhibitors (such as sulbactam, avibactam, and tazobactam), and harboring several plasmid replicon types, as well as disseminating widely through horizontal gene transfer (HGT) among bacteria, are reasons that highlight the emergence of New Delhi metallo-β-lactamase (NDM)-producing CR-KP strains. Several studies have indicated that IncX3, IncFII, and IncC are the predominant plasmid replicon types harboring the *bla*_*NDM*−1_ gene [4, 5]. Additionally, CR-KP isolates are generated through the acquisition of virulent or resistant plasmids that are commonly transferred among Gram-negative bacteria, particularly members of the *Enterobacteriaceae* family, which can lead to more severe infections [3, 6].

On the other hand, in September 2019, the health and clinicians' system faced a terrible medical challenge since emerging of severe acute respiratory syndrome coronavirus 2 (SARS-CoV-2). The identical symptoms and laboratory abnormalities between patients with SARS-CoV-2 pneumonia and community-acquired pneumonia resulted in using empirical antibiotics treatment; however, prolonged hospitalization, especially in an intensive care unit (ICU), and consumption of a high proportion of board-spectrum antibiotics led to raising MDR and extensively-drug resistant (XDR) bacteria [7, 8]. Therefore, the present study aimed to genomic analysis of a *bla*_*NDM*−1_ carrying CR-KP isolate from ICU admitted patients with SARS-CoV-2 in Sanandaj, west Iran.

## 2. Materials and Methods

### 2.1. Bacterial Isolation

The *K. pneumoniae* isolate was recovered in December 2022 from bronchoalveolar lavage (BAL) fluid of a 68 year-old male patient hospitalized in the ICU at Besat Hospital, a teaching hospital with 350 beds in Sanandaj, Iran. The isolate was identified as *K. pneumoniae* using standard biochemical tests, and confirmation was performed by amplifying the 16*S rRNA* gene [9].

### 2.2. Antimicrobial Susceptibility Testing

The CRKP-51 strain has sought to determine the minimum-inhibitory concentration (MIC) against 15 antibiotics using VITEK 2 (bioMérieux, Marcy l'Étoile, France). The antimicrobial agents tested were as follows amikacin (AMK), ampicillin (AMP), cefazolin (CFZ), cefepime (FEP), cefoxitin (FOX), ceftazidime (CAZ), ceftriaxone (CRO), ciprofloxacin (CIP), ertapenem (ETP), gentamicin (GEN), imipenem (IMP), levofloxacin (LVX), nitrofurantoin (NIT), piperacillin/tazobactam (TZP), tigecycline (TGC), and trimethoprim-sulfamethoxazole (SXT). The interpretation was performed regarding CLSI 2023 guidelines [10].

### 2.3. DNA Extraction and Whole-Genome Sequencing and De Novo Assembly

The bacterial DNA was extracted using a FavorPrep™ Tissue Genomic DNA Extraction Mini Kit (Favorgen, Taiwan). The Genomic library preparation was performed using the Illumina Novaseq6000 platform, and 150 bp paired-end reads were generated with an insert size of around 350 bp (Illumina Inc., San Diego, CA). The short reads were trimmed, and de novo assembly was performed using CLC workbench 20.0.

### 2.4. Post De Novo Assembly

The obtained contigs were assessed further using different online website tools as follows: using DFAST (https://dfast.ddbj.nig.ac.jp/) to demonstrate the genomic statistic information of assembled contigs such as genome length, GC content, N50, and number of coding sequences (CDSs), rRNA and tRNA as well as gene annotation. The contigs were categorized as chromosomal and extra-chromosomal using mlplasmids—version 1.0.0 (https://sarredondo.shinyapps.io/mlplasmids/). Multilocus-sequence typing (MLST) was determined using MLST Server 2.0 (https://cge.cbs.dtu.dk/services/MLST/). Evaluation of plasmid incompatibility (Inc) was accomplished using PlasmidFinder 2.0 server (https://cge.cbs.dtu.dk/services/PlasmidFinder/). The antimicrobial resistance genes profile was determined using the antibiotic resistance database (CARD) (https://card.mcmaster.ca/analyze/rgi). Capsular typing (K-typing) and allelic were detected using the Kaptive web-tool (https://kaptive-web.erc.monash.edu/). Multiple circular alignments of plasmid carrying NDM-1 with other similar genomes was conducted and visualized using a CG view server (https://cgview.ca/).

### 2.5. Accession Number

This Whole Genome Shotgun project has been deposited at DDBJ/ENA/GenBank under the accession JARGDG000000000. The version described in this paper is version JARGDG010000000.

## 3. Results

### 3.1. Antimicrobial Susceptibility Testing

The isolated strain was resistant to all tested antibiotics except for TGC (MIC = 2 μg/mL). The highest resistance value was recorded against SXT, NIT, and TZP (MIC ≥ 320 μg/mL, MIC = 256 μg/mL, and MIC ≥ 128 μg/mL), respectively. Also, the isolate was identified as a carbapenemase-producing strain. The complete results are available in Table 1.

### 3.2. Chromosomal Characterization

#### 3.2.1. Genomics Properties

The analysis of the whole-genome purified contigs determined 174 contigs comprised of 5,906,552 bp genome. Totally, 5625 features were recorded, followed by 67 tRNAs, 3 rRNAs, and 2 CRISPR regions on the whole genome. 80 out of 174 contigs were identified as chromosomal contigs (total length = 5,144,305 bp, GC content = 57.5%, N50 = 113,131, No. of CDSs = 4773, No. of rRNA = 3, No. of tRNA = 66 and coding ratio = 88.0%).

#### 3.2.2. MLST and Capsular Typing

The CRKP-51 strain belonged to ST 15 (*gapA*: 1, *infB*: 1, *mdh*: 1, *pgi*: 1, *phoE*: 1, *rpoB*: 1, *tonB*: 1). Also, the *K. pneumoniae* capsular locus KL-112 was detected for the CRKP-51 strain, the *cps* locus length was 24,370 bp consisting of 18 open reading frames (ORFs) from *galF* to *ugd*. The allelic types were recorded as follows *wzc*-923 and *wzi*-23. The six identified genes on the 5′ and 3′ conserved ends were *galF*, *cpsACP*, *wzi*, *wza*, *wzb*, *wzc* and *ugd*, *manB*, *manC*, *gnd*, *wzx*, *wcuV*, respectively, with coverage of 100% and identity of > 99%.

#### 3.2.3. Antimicrobial Resistance Genes (ARGs)

The analysis of chromosomal contigs has revealed nine ARGs as follow: *bla*_SHV−28_ and *bla*_SHV−106_ (β-lactam resistance); *gyrA*, *parC* (quinolone resistance); *fosA* (fosfomycin resistance); *ramR* (tigecycline resistance); *oqxA*, *oqxB*, *ompK*36 (MDR).

### 3.3. Plasmid Data

**Figure 1:**
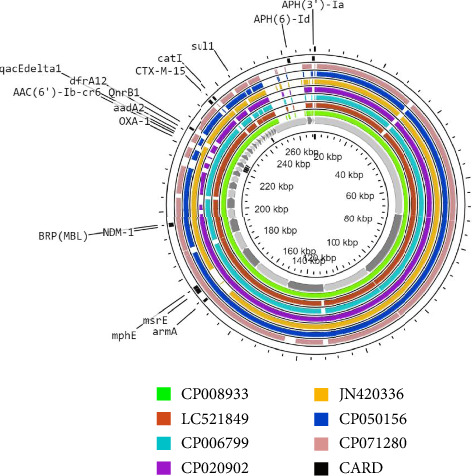
Circular alignment of Plasmid-1 with six highly similar NDM carrying plasmids including CP008933 as reference (Nepal), LC521849 (Japan), CP006799 (USA), CP020902 (Norway), JN420336 (Italy), CP050156 (Hong Kong), and CP071280 (Saudi Arabia). Alignment and illustration of multiresistance genes on plasmids was performed using (https://cgview.ca/) servers.

Totally four plasmids have determined among WGS data: in which Plasmid-1, as the biggest one, consisting of 269,316 bp length (CDSs = 278) belonged to the IncHI1B replicon type and harboring *bla*_*NDM*−1_, *armA*, *msrE*, *mphE*, *BRP* (MBL), *bla*_*OXA*−1_, *aadA*2, *dfrA*12, *qnrB*1, *bla*_*CTX*−*M*−15_, and *catl*. This is followed by Plasmid-2 (210,792 bp; CDSs = 184; belonged to IncFIB and IncFII), Plasmid-3 (178,789 bp; CDSs = 185; belonged to IncFIB), and Plasmid-4 (111,545 bp; CDSs = 133; belonged to IncFII and repB). In addition, Plasmid-3 and Plasmid-4 harbored the *tetD* and *dfrA*14, respectively. No resistance genes were detected for Plasmid-2.  Figure 1 illustrates the alignment results of Plasmid-1 with six highly similar containing the *bla*_*NDM*−1_ plasmid sequences. Also, multiresistance genes were determined on alignment plasmids of which explain the role of NDM harboring plasmid in development MDR, XDR, or even pan-drug-resistant (PDR) strains.

## 4. Discussion

During coronavirus disease of 2019 (COVID-19) pandemic ICU admissions were remarkably raised; it is estimated up to 5% patients with COVID-19 were admitted to ICU. This overcrowding and overusing antibiotics promote dissemination of MDR and XDR pathogens including *K. pneumoniae* [11]. Interestingly, emerging CR-KP isolates have been significantly reported in several investigations. For instance, in Italy, an increase in CR-KP frequency from 6.7% to 50% was reported between 2019 and 2020 [12]; as such increasing also documented by other study in New York [13]. This finding is consistent with an earlier systematic review and meta-analysis by Abubakar et al. which demonstrated an increase in the incidence of CR-KP following the COVID-19 pandemic compared to the period prior to the pandemic [14].

Notably, CR-KP are of significant concern due to their resistance to a wide range of antibiotics, including carbapenems and colistin. This resistance severely limits available treatment options resulting increasing rate of mortality [15]. The pooled prevalence of the mortality rate associated with CR-KP isolate infections ranged from 37.2% (95% CI, 33.1%–41.4%) to 42.1% (95% CI, 37.1%–47.3%) [16, 17]. Although the frequency of nosocomial infections caused by CR-KP isolates varies among conducted surveys due to factors such as sample size, geographic regions, study duration, and available resources, it is notable that the rate of nosocomial infections caused by CR-KP has increased over the last decade [18, 19].

CR-KP spreading is chiefly accomplished by a few successful clones; high-risk clonal (HiRC) lineages like ST11 and ST15 are as representatives of this phenomena [20]. The CRKP-51 strain isolated from an ICU-admitted patient with COVID-19 determined as ST15. Previous studies around the world, such as Spaine [21], France [22], China [23], and Brazil [24] have stated that *K. pneumonia* ST15 is one of the most relevant STs in nosocomial infections outbreaks. Furthermore, reviewing situation of antimicrobial resistance in ICUs has listed *K. pneumoniae* ST15 as a HiRC causes ICU outbreak during COVID-19 pandemic [25].

In this study, CRKP-51 revealed a variety of antibiotic resistance genes. Structurally, the majority of these ARGs are located on plasmids, which facilitate the potential for HGT. As hypothesized in other publications, ST15 is associated with the development of HGT and has a high propensity for the acquisition of resistance genes [9, 26, 27].

According to the available complete sequence in GenBank, 20 replicon types were identified for NDM carrying plasmids. Several studies have reported IncX3, IncA/C, IncFII, IncFIB, and IncFIA as predominant replicon types of *bla*_*NDM*−1_ carrying plasmids among *Enterobacteriaceae* family [28, 29]. However, in the present study *bla*_*NDM*−1_ carrying plasmid belonged to IncHI1B/IncFIB; also, co-existence of *bla*_*CTX*−*M*−15_ and *bla*_*NDM*−1_ genes on the same replicon types for CRKP-51 strain. Furthermore, the IncHI1B/IncFIB replicon type was associated with several ARGs, including *armA*, *msrE*, *mphE*, BRP (MBL), *bla*_*OXA*−1_, *aadA*2, *dfrA*12, *qnrB*1, and *cat*1. These findings align with a previous report by Shelenkov et al. which described ST512 *K. pneumoniae* isolates carrying IncHI1B/IncFIB plasmids harboring *bla*_*NDM*−1_ and other ARGs [30]. Further investigations confirmed harboring several ARGs along with *bla*_*NDM*_ has been [31, 32]. Thus, the high dissemination of bla_*NDM*_ genes among Gram-negative bacteria likely promotes MDR, XDR, and even PDR strains worldwide. This promotes a significant limitation in the available therapeutic options because there are not any β-lactamase inhibitors for preventing NDM activity, even though ceftazidime/avibactam, as well as aztreonam (as only active β-lactam agent against NDM variants) hydrolyses by CTX-M-15.

Although additional research is required to elucidate the full range of mobile genetic elements contributing to *blaNDM* gene spread and antimicrobial resistance development, inhibiting plasmid dissemination may offer a viable approach to mitigate bacterial resistance. This study was not designed to examine the broader distribution and characterization of CR-KP across Iran due to sample size limitations. Furthermore, conjugation assays are recommended to assess the potential for HGT among Gram-negative bacteria.

In summary, antimicrobial resistance has increased in ICUs mainly due to HiRC spread, which has played a significant role in the global spread of MDR bacteria. *K. pneumoniae* ST15 has recently taken attention as a HiRC associated with global dissemination of NDM enzyme, particularly among ICU-admitted patients. Also, genomic analysis of CRKP-51 demonstrated that NDM carrying plasmid promotes MDR strains through harboring other mediated multiresistance genes. In addition, there is a lack of knowledge about the impact of overusing antimicrobial agents during COVID-19 pandemic, so monitoring local epidemiology is essential for antimicrobial stewardship programs.

## Figures and Tables

**Table 1:** Antimicrobial susceptibility of CRKP-51 strain.

Susceptibility information					
Antimicrobial	MIC	Interpretation	Antimicrobial	MIC	Interpretation
Amikacin	≥ 64	R	Ertapenem	≥ 8	R
Ampicillin	≥ 32	R	Gentamycin	≥ 16	R
Cefazoline	≥ 64	R	Imipenem	≥ 16	R
Cefepime	≥ 64	R	Levofloxacin	≥ 8	R
Cefoxitin	≥ 64	R	Nitrofurantoin	256	R
Ceftazidime	≥ 64	R	Piperacillin/tazobactam	≥ 128	R
Ceftriaxone	≥ 64	R	Tigecycline	≥ 2	S
Ciprofloxacin	≥ 4	R	Trimethoprim/sulfamethoxazole	≥ 320	R

Abbreviations: R, resistant; S, sensitive.

## Data Availability

The data that support the findings of this study are available from the corresponding author upon reasonable request.

## Nomenclature

*K. pneumoniae*: *Klebsiella pneumonia*
MDR: Multidrug resistant
XDR: Extensively-drug resistant
PDR: Pan-drug resistant
BAL: Bronchoalveolar lavage
ST: Sequence type
ARGs: Antimicrobial resistance genes
COVID-19: Coronavirus disease of 2019
ICU: Intensive care unit
HiRC: High-risk clonal
